# Expression and Biological Activity Analysis of Recombinant Fibronectin3 Protein in *Bacillus subtilis*

**DOI:** 10.3390/biotech14030051

**Published:** 2025-06-23

**Authors:** Chaozheng Lu, Guangxin Xu, Yin Tian, Zhiwei Yi, Xixiang Tang

**Affiliations:** Key Laboratory of Marine Genetic Resources, Third Institute of Oceanography, Ministry of Natural Resources, Xiamen 361005, China; luchaozheng@tio.org.cn (C.L.); xuguangxin@tio.org.cn (G.X.); tianyin@tio.org.cn (Y.T.)

**Keywords:** recombinant fibronectin3 protein, *Bacillus subtilis*, cell migration, cell adhesion

## Abstract

Fibronectin (FN), a primary component of the extracellular matrix (ECM), features multiple structural domains closely linked to various cellular behaviors, including migration, spreading, adhesion, and proliferation. The FN3 domain, which contains the RGD sequence, is critical in tissue repair because it enables interaction with integrin receptors on the cell surface. However, the large molecular weight of wild-type FN presents challenges for its large-scale production through heterologous expression. Therefore, this study focused on cloning the FN3 functional domain of full-length FN for expression and validation. This study selected *Bacillus subtilis* as the expression host due to its prominent advantages, including efficient protein secretion, absence of endotoxins, and minimal codon bias. The recombinant vector pHT43-FN3 was successfully constructed through homologous recombination technology and transformed into *Bacillus subtilis* WB800N. The FN3 protein was successfully expressed after induction with IPTG. Following purification of the recombinant FN protein using a His-tag nickel column, SDS-PAGE analysis showed that the molecular weight of FN3 was approximately 27.3 kDa. Western blot analysis confirmed the correct expression of FN3, and the BCA protein assay kit determined a protein yield of 5.4 mg/L. CCK8 testing demonstrated the good biocompatibility of FN3. In vitro cell experiments showed that FN3 significantly promoted cell migration at a 20 μg/mL concentration and enhanced cell adhesion at 10 μg/mL. In summary, this study successfully utilized *Bacillus subtilis* to express the FN3 functional domain peptide from FN protein and has validated its ability to promote cell migration and adhesion. These findings not only provide a strategy for the expression of FN protein in *B. subtilis*, but also establish an experimental foundation for the potential application of FN3 protein in tissue repair fields such as cutaneous wound healing and cartilage regeneration.

## 1. Introduction

Fibronectin (FN), a primary component of the extracellular matrix (ECM), was initially discovered and termed a cold-insoluble protein due to its characteristic precipitation upon exposure to cold temperatures [1]. FN is a high-molecular-weight dimeric glycoprotein widely present in animal tissues and tissue fluids, with a full-length molecular weight of approximately 440 kDa and containing multiple functional domains [2]. Among its key functional domains, FN1, FN2, and FN3 facilitate fibrin binding, forming a scaffold structure that promotes cell migration [3]. FN is a critical regulator of various cellular behaviors such as migration, adhesion, proliferation, and invasion [4,5,6]. In wound healing research, it has been discovered that FN can serve as a nonspecific opsonin, enhancing the function of the immune system to eliminate bacteria and necrotic tissue from the wound site [7]. FN also facilitates hemostasis by forming a fibrin–fibronectin network [8]. Additionally, FN interacts with integrins on the cell membrane, guiding the migration of epithelial cells and fibroblasts to wound areas, thereby accelerating the healing process [9,10]. Furthermore, FN serves as a natural cell adhesion factor [11], and it promotes the adhesion of osteoblasts, enabling them to exhibit a more complete cytoskeletal morphology [12]. The co-application of FN with platelet-derived growth factor-BB (PDGF-BB) can enhance cell and tissue survival rates under stress [13]. When combined with biomaterials, nanofiber scaffolds loaded with FN can facilitate the migration and adhesion of dental mesenchymal cells, thus promoting dental pulp regeneration [14]. In disease prediction and diagnosis, fetal FN influences the interaction between uterine detachment and fetal membranes, a significant indicator for predicting preterm birth, thereby reducing mortality associated with preterm birth [15]. Therefore, FN exhibits functionality across many domains and presents potential application prospects in biomedicine and tissue engineering. However, due to the significant molecular weight of full-length FN protein, heterologous expression using genetic recombination technology still faces technical challenges, resulting in limited clinical application [16]. Hence, cloning and expressing the active functional regions of full-length FN is an effective strategy to address these challenges.

*Bacillus subtilis*, a soil-dwelling bacterium, has been extensively investigated as a model organism for Gram-positive bacteria. In addition to *Escherichia coli*, *B. subtilis* is also a commonly used host for recombinant protein production, as it exhibits rapid growth, high cell density, and minimal codon bias, facilitating efficient expression of heterologous proteins [17]. Importantly, unlike *E. coli*, which contains a lipopolysaccharide, known as an endotoxin, that can cause fever in humans and other mammals, *B. subtilis* is devoid of such endotoxin and is recognized as a safe microorganism by the US Food and Drug Administration (FDA) [18]. Therefore, from a safety perspective, *B. subtilis* is more advantageous than *E. coli* for protein production in the pharmaceutical, cosmetic, and food industries [19]. Studies have demonstrated that *B. subtilis* possesses only a single-layer cell membrane, facilitating the secretion of recombinant proteins across the membrane into the extracellular space, thereby significantly reducing downstream purification coats [20]. Additionally, *B. subtilis* can eliminate misfolded or incompletely synthesized proteins during protein synthesis [21]. Although this occasionally results in lower levels of expression, stable high-level protein expression can be achieved through combinations of strong promoters, transcriptional terminators, and various translational/secretion signals [22,23]. However, the extracellular proteases secreted by *B. subtilis* can limit the expression of heterologous proteins, leading to low protein yields [24]. To address this issue, researchers have successfully constructed *B. subtilis* WB800N by knocking out eight protease genes in *B. subtilis* 168, effectively resolving the problem of secreted protein hydrolysis [24,25]. Research has shown that the expression level of nattokinase in *B. subtilis* WB800N is higher than in other extracellular protease-deficient *B. subtilis* expression systems when using the same promoter, such as WB700 and WB600 [26]. Furthermore, by introducing the θ-replication mode of a native plasmid and replacing the original *groE* operon with the *lac* operon, researchers not only resolved the instability of the original vector but also rendered the induced expression process more efficient and controllable. Thus, using *B. subtilis* to express the active functional domains of FN enables the efficient production of recombinant proteins while simplifying the downstream protein purification process.

Numerous studies have investigated the functions of various domains within FN, focusing on the FN3 domain containing the arginine–glycine–aspartic acid (RGD) sequence [27]. This specific region promotes the binding of FN to the integrin α5β1 receptor on the cell surface, thereby facilitating cell adhesion and migration [28,29]. However, there is currently limited focus on the expression and functional validation of individual FN domains. In this study, *B. subtilis* WB800N was selected as the expression host. The FN3 domain containing the RGD sequence was cloned from full-length FN and inserted into the pHT43 expression vector, enabling the construction of a stable extracellular expression system in *B. subtilis*. This system successfully expressed the recombinant FN3 protein. The recombinant FN3 protein was purified using a His-tag nickel column, and its correct expression was verified through SDS-PAGE and Western blot analyses. The CCK-8 assay illustrated the good biocompatibility of FN3. The biological activity of FN3 was preliminarily analyzed using cell scratch and adhesion assays, demonstrating that FN3 significantly promotes L-929 cell migration and adhesion. These findings lay the groundwork for the application potential of FN3 in tissue engineering, especially in wound repair and bone regeneration.

## 2. Materials and Methods

### 2.1. Material

QuickCut *Bam*H I was purchased from Takara (Beijing, China). The Seamless cloning kit, high-fidelity enzyme, Taq enzyme, and DNA Marker were obtained from Vazyme (Nanjing, China). The plasmid extraction and DNA gel recovery kits were sourced from AG (Changsha, China). The protein marker, His-tag nickel column, SDS-PAGE gel, Cell Counting Kit-8 (CCK-8, C0038), BCA Protein Concentration kit (P0012), ECL chemiluminescence kit (P0018M), and Luria–Bertani (LB) medium were purchased from Beyotime (Shanghai, China). IPTG (Isopropyl β-D-thiogalactoside, I8070), Ampicillin (Amp, A8180), Chloramphenicol (Cm, C8080), and Bovine albumin (BSA, A8020) were acquired from Solarbio (Beijing, China). A 10 kDa protein ultrafiltration tube (UFC9010) was brought from Merck (DE, Darmstadt, Germany). Roswell Park Memorial Institute-1640 basic medium (RPMI-1640), Penicillin–Streptomycin (PS), Phosphate-Buffered Saline (PBS), and trypsin were brought from Procell (Wuhan, China). Fetal Bovine Serum (FBS) was obtained from ABW (Shanghai, China). Crystal violet was purchased from Macklin (Shanghai, China). His-tag antibody (Anti-His) and HRP-conjugated Goat Anti-Mouse IgG secondary antibody were procured from Servicebio (Wuhan, China).

### 2.2. Construction of the Recombinant Plasmid pHT43-FN3

The upstream and downstream primers for the recombinant FN3 gene were designed using SnapGene (4.1.9) software, with sequences homologous to the *Bam*H I restriction site incorporated. DH5α-pET22b-FN3 is a strain previously designed and maintained in the laboratory. Specifically, the DH5α-pET22b-FN3 strain was activated to extract plasmid DNA, which was then used as a template for PCR amplification according to the instructions provided with the high-fidelity enzyme system. Simultaneously, the pHT43 vector was subjected to single digestion using *Bam*H I. After completing PCR and restriction digestion reactions, agarose gel electrophoresis was performed. The target bands were recovered using a DNA gel extraction kit, and the DNA concentration was determined. The linearized vector and target gene amounts were calculated based on the instructions from the seamless cloning kit before the reaction. The recombinant product was transformed into DH5α competent cells, which were then plated on LB agar containing 1% Amp and incubated overnight. Single colonies were selected and inoculated into LB medium containing 1% Amp, followed by overnight incubation at 37 °C. Plasmids were extracted, and colony PCR was performed for verification. The verified recombinant plasmids were sent to BioSune (Shanghai, China) for sequencing. The recombinant plasmid, confirmed by sequencing, was named pHT43-FN3. The primer sequences of FN3 gene amplification and colony PCR are detailed in Table 1.

### 2.3. Transformation and Expression of FN3 Recombinant Plasmid

The recombinant plasmid pHT43-FN3 was transformed into *B. subtilis* WB800N cells using the Spizizen method to construct the recombinant strain WB800N-pHT43-FN3. The positive strains were identified using colony PCR. The recombinant strain WB800N-pHT43-FN3 and the control group WB800N-pHT43 were inoculated into 5 mL of LB medium containing 25 μg/mL Cm and incubated overnight at 37 °C with shaking at 180 rpm. The culture was subsequently transferred to 500 mL of LB medium with 25 μg/mL Cm at a final volume ratio of 1% and incubated at 37 °C until the OD600 reached 0.6 to 0.8. Protein expression was induced overnight by adding 0.1, 0.5, and 1 mM of IPTG to the culture medium at 25 °C and 37 °C with shaking at 150 rpm. After induction, the cultures were centrifuged at 8000 rpm for 15 min at 4 °C to separate the supernatant and cell pellet. The cell pellet before induction, the cell pellet after induction, and the supernatant after induction were collected for SDS-PAGE analysis to detect protein expression. The protein 3D structure of the FN3 protein was determined utilizing the SwissModel web platform (http://swissmodel.expasy.org/). Meanwhile, the functional domains within the FN3 protein were identified through an analysis conducted on the SMART database (https://smart.embl.de/).

### 2.4. Purification and Identification of the Recombinant FN3 Protein

Based on the characteristics of the *B. subtilis* expression system, the FN3 proteins secreted by the system were extracted by ammonium sulfate precipitation. After incubating at 4 °C for 30 min, the protein precipitate was collected by centrifugation at 8000 rpm for 15 min at 4 °C. The precipitate was then dissolved in a non-denaturing lysis buffer and filtered through a 0.45 μm membrane for further use. The protein solution was mixed with a His-tag nickel column and incubated on a vertical mixer at 4 °C for 1 h, with rotating conditions to the binding efficiency of the FN3 protein to the nickel column. Subsequently, a washing buffer containing 3 mM imidazole was used to remove nonspecifically adsorbed impurities. Finally, an elution buffer containing 50 mM imidazole was applied to elute and collect the target protein. The flow-through, wash, and elution fractions were analyzed by SDS-PAGE gel electrophoresis to assess protein purification. The target protein fraction was desalted using a 10 kDa protein ultrafiltration tube, with PBS as the replacement buffer. The protein concentration of the desalted solution was determined using a BCA protein assay kit to calculate the protein yield. The concentration was adjusted to 1 mg/mL, filtered through a 0.22 μm membrane, and stored at −20 °C.

### 2.5. Western Blotting

A Western blot was analyzed using Anti-His and HRP-conjugated Goat Anti-Mouse IgG antibodies as the secondary antibody to confirm the identity of the purified protein. Specifically, the purified FN3 protein was subjected to SDS-PAGE electrophoresis for protein separation. The proteins were transferred onto a polyvinylidene fluoride (PVDF) membrane and blocked with 5% non-fat milk for 1 h at room temperature. The PVDF membrane was then incubated in a prepared dilution of Anti-His antibody (1:1000) for 1 h at room temperature. After washing the PVDF membrane three times, it was transferred to Goat anti-Mouse IgG secondary antibody (1:3000) and incubated for 1 h at room temperature. Finally, the protein bands were detected using an ECL chemiluminescent reagent.

### 2.6. Effect of FN3 on the Proliferation of L-929 Cells

L-929 fibroblast cells (L-929) were obtained from the China Center for Type Culture Collection (GDC0034, CCTCC, Wuhan, China). L-929 was cultured in RPMI-1640 supplemented with 10% FBS and 1% PS under 37 °C and 5% CO_2_. Once the cells reached 80% confluency, L-929 cells were digested with 0.25% trypsin and resuspended as a cell suspension. The cell density was adjusted to 1 × 10^5^ cells/mL, and 100 μL of cell suspension was seeded in 96-well plates. After 24 h, the culture medium of the experimental group was refreshed with a complete medium supplemented with FN3 at concentrations of 5, 10, 20, 50, and 100 μg/mL. In contrast, the control group cells were maintained in an equal volume of fresh medium without FN3 and incubated at 37 °C in a 5% CO_2_ environment. Medium renewal was performed on days 1, 3, and 5 of cell culture. At each time point, 100 μL of fresh medium containing 10 μL of CCK-8 reagent was added to each well of the 96-well plate, followed by a 2 h incubation at 37 °C. Subsequently, absorbance at 450 nm was measured using an enzyme maker (Thermo Fisher Scientific, Waltham, MA, USA). Six independent replicates were performed for each experimental condition.

### 2.7. Effect of FN3 on the Migration of L-929 Cells

Before the cells were inoculated, 5 parallel lines were drawn at the bottom of each hole of the sterile 6-well plate with a marker pen to locate the fixed positions to be photographed. The cell suspensions were prepared using a method previously described. After adjusting the cell density to 3 × 10^5^ cells/mL, 2 mL of cell suspension was seeded into 6-well plates and incubated at 37 °C and 5% CO_2_. Once the cells adhered, a 200 μL pipette tip was used to perform a linear scratch in a 6-well plate. Subsequently, the wells were rinsed three times with PBS. In the experimental groups, media containing 1% FBS with varying concentrations of FN3 (5, 10, 20, 50, 100 μg/mL) were added, while the control group received media with 1% FBS but without FN3. The area of the scratch region was observed and photographed at 0, 12, 24, and 48 h under an inverted microscope (Leica, DE) and analyzed using Image J (1.54g) software. The cell migration experiment was repeated three times independently for each group.

### 2.8. Effect of FN3 on the Adhesion of L-929 Cells

The 96-well plate was coated with 100 μL of PBS solutions containing 0, 5, 10, 20, 50, and 100 μg/mL of FN3 and incubated at 4 °C overnight. After discarding the coating solution, 200 μL of heat-denatured 1% BSA was added to block the plates for 1 h at 37 °C, and the wells were rinsed with PBS three times. The cell suspensions were prepared in serum-free media using a method previously described. After adjusting the cell density to 1 × 10^5^ cells/mL, 100 μL of cell suspension was seeded into 96-well plates and incubated at 37 °C and 5% CO_2_ for 2 h. Subsequently, unattached cells were washed away using 37 °C pre-warmed PBS. The cells were fixed with 4% paraformaldehyde for 15 min, rinsed twice with PBS, and stained with 200 μL of 0.1% crystal violet for 20 min. After staining, the residual crystal violet was washed away with PBS. The cells were observed under an inverted microscope, and images were recorded. The cell adhesion experiment was repeated three times independently for each group.

### 2.9. Statistical Analysis

Statistical analysis of all experimental results was conducted with GraphPad Prism 9.0. Quantitative measurements are expressed as the arithmetic mean with standard deviation (mean ± SD). Group comparisons were evaluated through a one-way ANOVA. Statistical significance was denoted as follows: * *p* < 0.05, ** *p* < 0.01, *** *p* < 0.001, and **** *p* < 0.0001. Non-significant differences were marked as ns *p* > 0.05.

## 3. Results and Discussion

### 3.1. Construction and Identification of the pHT43-FN3 Vector

**Figure 1:**
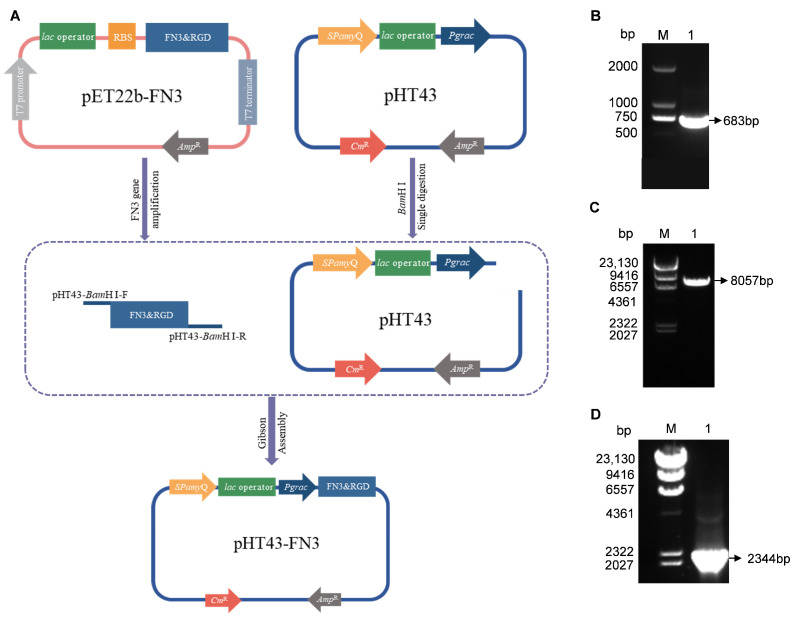
Construction process and PCR verification of pHT43-FN3. (**A**) Preparation of the recombinant plasmid pHT43-FN3. (**B**) PCR amplification of the FN3 fragment. (**C**) Single-enzyme digestion of the pHT43 vector. (**D**) Colony PCR fragments of the pHT43-FN3 recombinant strain.

Figure 1A illustrates the preparation process of pHT43-FN3 using homologous recombination technology. The pET22b-FN3 plasmid was used as a template, with pHT43-*Bam*H I-F as the upstream primer and pHT43-*Bam*H I-R as the downstream primer for PCR amplification; the recovered product fragment was 683bp (Figure 1B). Following single-enzyme digestion of pHT43, the purified product yielded a linearized vector of 8057 bp (Figure 1C). The recombinant FN3 fragment and the linearized pHT43 vector were subjected to homologous recombination and transformed into DH5α competent cells. Single colonies were selected and cultured, and plasmid DNA was extracted. Subsequently, colony PCR detection was performed using pHT43-FN3-F as the upstream primer and pHT43-FN3-R as the downstream primer, yielding a fragment of 2344 bp (Figure 1D). The above experimental results are all consistent with the expectations. The identified positive recombinant plasmid was sequenced and compared to the designed target sequence in SnapGene software, showing 100% sequence identity. This indicates the successful construction of the recombinant plasmid pHT43-FN3.

### 3.2. Induced Expression of FN3 and Preliminary Optimization of Conditions

**Figure 2:**
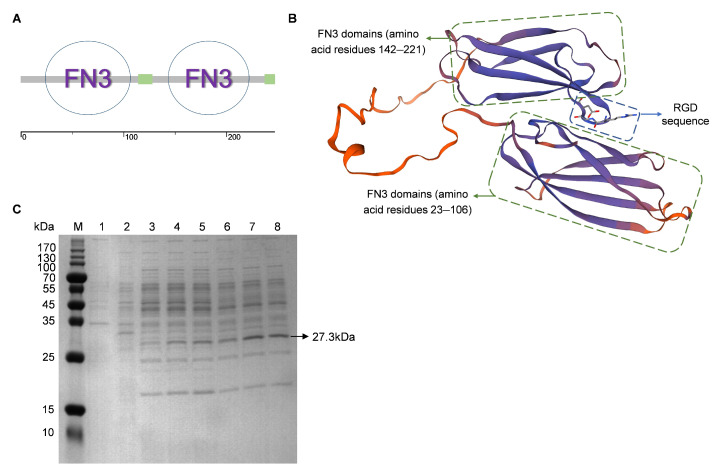
Bioinformatics prediction of FN3 and optimization of fermentation conditions. (**A**) Functional domain prediction of recombinant FN3. (**B**) Structural prediction of recombinant FN3. (**C**) SDS-PAGE electrophoresis of pellet and supernatant from recombinant *B. subtilis* before and after induction. M: protein marker; 1: bacterial culture before induction; 2: bacterial cells after induction; 3–5: fermentation supernatants at 28 °C with IPTG induction concentrations of 0.1, 0.5, and 1 mM in sequence; 6–8: fermentation supernatants at 37 °C with IPTG induction concentrations of 0.1, 0.5, and 1 mM in sequence.

Structural prediction using an online software tool revealed the presence of two FN3 structural domains in the recombinant FN3 protein, located at amino acid residues 23–106 and 142–221 (Figure 2A). Figure 2B depicts the predicted structure of FN3, with the RGD sequence located within one of its functional domains. The expression of FN3 from a recombinant *B. subtilis* strain was analyzed by SDS-PAGE electrophoresis. As shown in Figure 2C, a specific protein band appeared at approximately 27.3 kDa in the supernatant of the recombinant strain fermentation broth after IPTG induction, which was consistent with the expected results and provided initial evidence of FN3 expression. Analysis of lanes 3–8 in Figure 2C revealed that at the same temperature, increasing the concentration of IPTG to 0.5 mM had little effect on protein expression levels. However, at the same IPTG concentration, an induction temperature of 37 °C was superior to 28 °C. Therefore, subsequent FN3 expressions were carried out under conditions of 0.5 mM IPTG and 37 °C.

### 3.3. Purification and Desalination of Recombinant FN3

**Figure 3:**
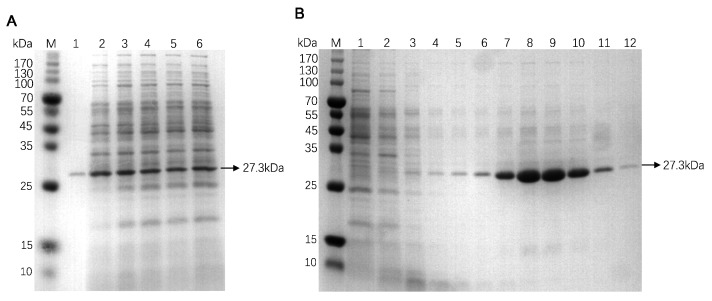
SDS-PAGE electrophoretogram of recombinant FN3 after ammonium sulfate precipitation and purification. (**A**) The fermentation supernatant was subjected to ammonium sulfate precipitation. M: protein marker; lanes 1–6: fermentation supernatants precipitated with 20%, 30%, 40%, 50%, 60%, and 70% ammonium sulfate, respectively. (**B**) The target protein was purified by a His-tag nickel column. M: protein marker; 1: flow-through fraction; 2–4: the 1st–3rd tubes of the eluent containing impurity proteins eluted with 3 mM imidazole in sequence; 5–12: The 1st–8th tubes of the eluent containing the target protein eluted with 50 mM imidazole in sequence.

The supernatant was concentrated by ammonium sulfate solution through a 20% to 70% saturation range to determine the optimal concentration for ammonium sulfate precipitation. As shown in Figure 3A, the results indicate that FN3 can be completely precipitated at a 30% ammonium sulfate concentration. However, with the increasing concentration of ammonium sulfates, the concentration of contaminant proteins also rises, which may lead to insufficient dissolution of ammonium sulfate. As shown in Figure S1, direct application of the post-fermentation supernatant to His-tag nickel column affinity chromatography resulted in significantly reduced levels of specifically bound protein and lower yields of purified protein. This made it challenging to achieve high concentrations of FN3 protein. The observed phenomenon may be attributed to the elevated salt concentration in the LB medium, which potentially disrupted the binding efficiency of the target protein to the nickel column, thereby reducing the overall purification efficiency. In Figure 3B, lane 1 demonstrates that the flow-through collected during the purification process using a His-tag nickel column contained almost no target protein. This suggests that FN3 exhibits a strong binding affinity to the nickel column, enabling effective purification of the target protein through the use of wash and elution buffers. The use of 3 mM imidazole effectively eluted contaminant proteins in the early stages, but as the elution volume increased, a small amount of FN3 may have also been eluted. It was found that FN3 was mainly eluted in tubes 3–6 when 50 mM imidazole was used to elute the target protein. The protein was then desalted using a 10 kDa ultrafiltration tube, and the protein concentration was determined using a BCA protein concentration detection kit. The final yield of FN3 was calculated to be 5.4 mg/L.

### 3.4. Identification of FN3 by Western Blot

**Figure 4:**
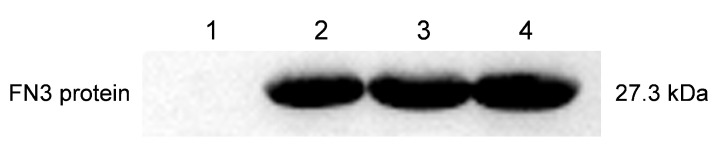
Western blot of recombinant FN3 protein. 1: empty vector negative control; 2–4: purified FN3 protein.

In the purification experiment of the FN3 protein, the expected target band was obtained through SDS-PAGE electrophoresis. However, further verification was needed before functional evaluation to confirm whether the purified protein is FN3. A Western blot was carried out using Anti-his and Goat Anti-Mouse IgG antibody as a secondary antibody to ensure the reliability of subsequent experiments because the recombinant FN3 was tagged with a His-tag. As shown in Figure 4, a distinct protein band at approximately 27.3 kDa was observed, consistent with the expected molecular weight of FN3, thereby confirming the identity of the expressed protein as FN3.

### 3.5. CCK8 Assay of the Effect of FN3 on L-929 Cell Proliferation

**Figure 5:**
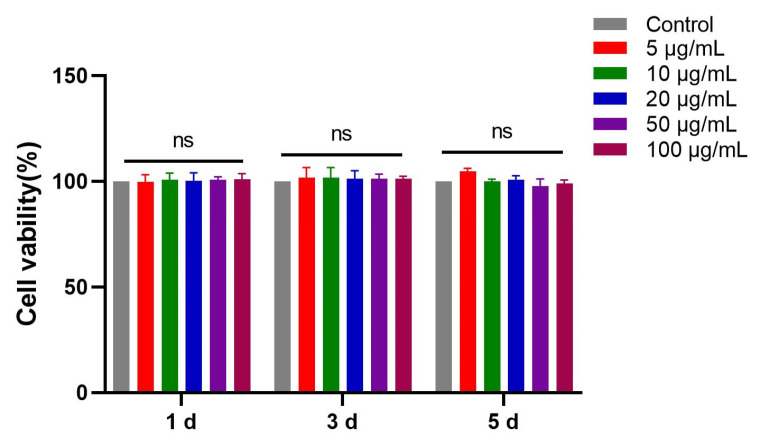
FN3 induces concentration-dependent effects on the viability of L-929 cells. ns *p* > 0.05 denotes a non-significant difference between the experimental and control groups, *n* = 6.

The effect of FN3 on the proliferation of L-929 cells was evaluated using the CCK-8 assay. As shown in Figure 5, the results indicated that FN3 at various concentrations did not significantly affect the proliferation of L-929 cells at any time. Notably, FN3 did not exhibit overt toxic effects on L-929 cells, even at high concentrations. It has been found that Chitosan-TiO2 scaffolds exhibit poor biocompatibility. However, incorporating FN significantly enhances both the biocompatibility of the scaffolds and the in vitro therapeutic effects [30]. Previous research on applying FN-loaded nano scaffolds in dental pulp regeneration has demonstrated that adding FN enhances the tissue regeneration effect of biomaterials and improves their biocompatibility [14]. Based on the experimental results, these findings provide a strong foundation for the potential application of FN3 in subsequent biomaterials research.

### 3.6. Effect of FN3 on the Scratch Assay of L-929 Cells

**Figure 6:**
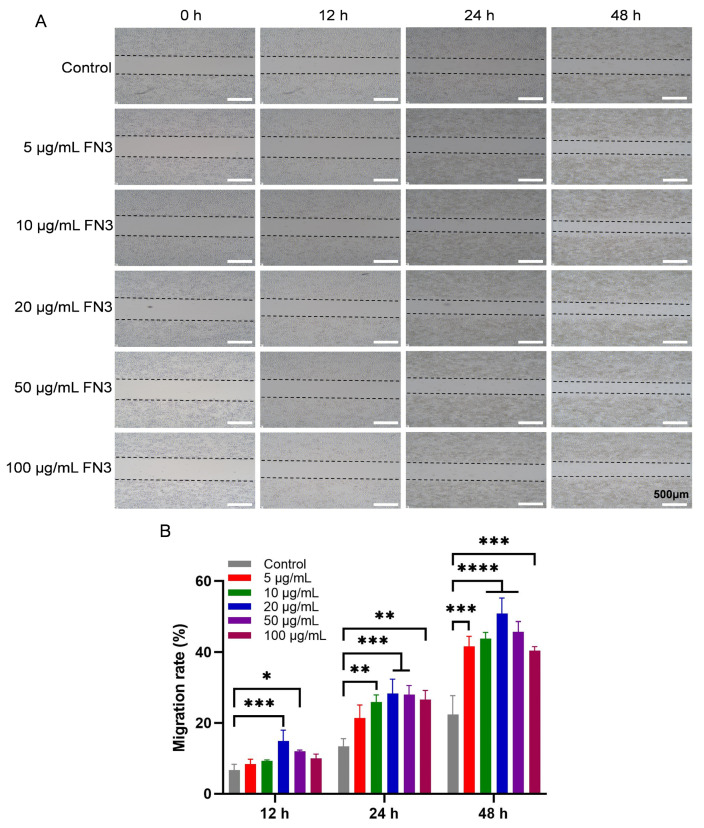
Effect of FN3 on the migration of L-929 cells. (**A**) The migration area of L-929 cells at different time points (×200). (**B**) The relative quantitative assessment of the migration area in L-929. * *p* < 0.05 shows a significant difference between the experimental and control groups; ** *p* < 0.01, *** *p* < 0.001, **** *p* <0.0001 show a highly significant difference between the experimental and control groups, *n* = 3.

The cell scratch assay is a methodological approach to assess cell migration ability and repair rate, resembling an in vitro wound healing model [31]. In this experiment, the effect of FN3 on the L-929 cell migration ability was evaluated by the cell scratch assay and migration experiment. The scratch areas were recorded at 0, 12, 24, and 48 h using images captured under an inverted microscope and analyzed with Image J software. Figure 6 illustrates the results of FN3 on the migration of L-929 cells. At 12 h, the migration area of the FN3 experimental groups at concentrations of 20 μg/mL and 50 μg/mL showed significant differences compared to the control group. After 24 h of culture, the healing rate of the FN3 experimental groups at both 20 μg/mL and 50 μg/mL concentrations was significantly higher than that of the control group. By 48 h, the scratch repair rate of all experimental groups at various concentrations was higher than that of the control group, with the experimental group treated at a concentration of 20 μg/mL demonstrating the best scratch repair effect. FN can activate focal adhesion kinase (FAK) through binding with integrin α5β1, inducing the formation of the FAK-Src complex and thereby participating in the promigratory signaling cascades [32]. This allows FN to play a direct role in promigratory mechanotransduction. Furthermore, the FAK signaling pathway also involves Src family kinases (SFKs) and colony-stimulating factor 1 receptor (CSF-1R) [33]. This was observed in murine macrophages and human macrophages. Therefore, it can be inferred that the RGD sequence in recombinant FN3 may specifically bind to α5β1, inducing the formation of FAK-Scr to promote the migration behavior of L-929 cells.

### 3.7. Crystal Violet Assay of the Adhesion Effect of FN3 on L-929 Cells

**Figure 7:**
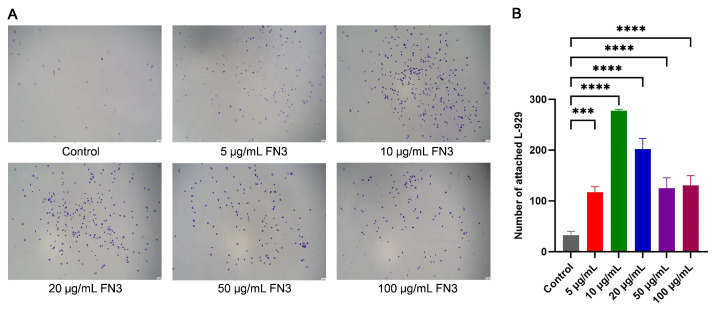
Detection of the adhesion effect of recombinant FN protein on L-929 cells by crystal violet staining. (**A**) cell images stained with crystal violet (×200). (**B**) Quantification of adhered cells. *** *p* < 0.001, **** *p* < 0.0001 indicate a highly significant difference between the experimental and control groups, *n* = 3.

This study verified the adhesion effect of FN3 protein on L-929 cells through crystal violet staining. The cells appeared rounded and did not fully adhere to the wall without the addition of FN3, resulting in fewer adherent cells. Conversely, the cells displayed a more complete cellular morphology when FN3 was applied. The total number of cells was counted in six randomly selected fields. It was observed that the number of adhered cells in the FN3 experimental groups at different concentrations showed varying degrees of difference compared to the control group, with the 10 μg/mL FN3 exhibiting the highest number of adhered cells (Figure 7). FN plays a profound role as a biological glue within the ECM, serving as one of the critical components for cell adhesion [34]. The binding of FN to integrin receptors is closely associated with the RGD sequence, providing anchor points for cells within the ECM [35]. Additionally, integrin α5β1 expressed by fibroblasts and integrin αVβ3 expressed by endothelial cells play significant roles in mediating cell–ECM adhesion [36]. FN has been shown to promote mesenchymal stem cell adhesion on the material surface, thereby supporting large-scale cell culture [37]. Studies have demonstrated that a composite scaffold prepared via conjugation of FN with chitosan/nano-β-tricalcium phosphate (β-TCP) exhibits excellent cell adhesion properties and results in an increase in cell number [38]. These findings suggest that the FN3 protein may interact with integrins in L-929 cells, enhancing adhesion and proliferation on the material surface. This is consistent with the experimental observations of adhesion. Interestingly, FN3 at a concentration of 10 μg/mL significantly enhances cell–matrix interactions within a short timeframe. This effect may be attributed to conformational adjustments occurring during FN3 recombinant expression, which expose additional binding sites and amplify their biological activity.

## 4. Conclusions

FN plays a critical role in tissue repair by regulating cellular behavior, particularly in mediating cell adhesion and migration, and has attracted significant attention. However, the current commercial full-length FN protein remains prohibitively expensive, and its large molecular weight results in low heterologous expression efficiency, thereby limiting its potential as a bioactive component in biomaterial development. This study focuses on the expression and functional validation of the FN3 functional domain. By cloning the FN3 domain containing the RGD sequence from full-length FN, we successfully obtained the recombinant FN3 sequence. The expression strain *B. subtilis* WB800N/pHT43-FN3 was constructed, enabling efficient secretory expression of the FN3 protein. The expressed FN3 protein has a molecular weight of approximately 27.3 kDa, and its correct expression was confirmed by Western blot analysis. Through nickel affinity chromatography, FN3 protein with a purity of >90% was successfully purified, achieving a 5.4 mg/L yield. However, there is still room for improvement in FN3 expression levels, which could be further enhanced by optimizing fermentation media and conditions. In vitro, CCK-8 assays confirmed the good biocompatibility of recombinant FN3. Cell scratch and adhesion assays demonstrated that FN3 significantly enhanced the migration capacity of L-929 cells at a concentration of 20 μg/mL and improved cell adhesion to the substrate at 10 μg/mL. Although in vitro cell experiments have initially verified the biological activity of FN3, its role in complex in vivo microenvironments remains to be validated through animal models. In conclusion, this study successfully utilized *Bacillus subtilis* to express the FN3 protein, thereby providing a strategy for the heterologous expression of large FN proteins. Additionally, FN3 exhibits excellent biocompatibility and demonstrates significant capabilities in promoting cell migration and adhesion, thereby demonstrating immense application potential in tissue engineering fields such as wound repair and bone regeneration.

## Figures and Tables

**Table 1:** Primer sequences for FN3 gene amplification and colony PCR.

Primer Name	Sequence (5′–3′)
pHT43-FN3-*Bam*H I-F	CAAAAACATCAGCCGTAGATATGACCCCGTCTCAGCCG
pHT43-FN3-*Bam*H I-R	GGACGTCGACTCTAGAGATTAGTGGTGGTGGTGGTGATG
pHT43-FN3-F	GTAAAACGACGGCCAGT
pHT43-FN3-R	CGGGGACGTCGACTCTA

## Data Availability

The original contributions presented in this study are included in the article/Supplementary Materials. Further inquiries can be directed to the corresponding authors.

## Supplementary Materials

The following supporting information can be downloaded at https://www.mdpi.com/article/10.3390/biotech14030051/s1, Figure S1: The fermentation supernatant underwent protein purification via nickel affinity chromatography.
