# Supplementary material for: Expression and Biological Activity Analysis of Recombinant Fibronectin3 Protein in Bacillus subtilis
Source: BioTech (Basel). 2025 Jun 23;14(3):51. doi: 10.3390/biotech14030051 (PMC12286206; doi:10.3390/biotech14030051)
Supplement: Supplementary file 1 [file biotech-14-00051-s001.zip › biotech-3688005-supplementary.pdf]

Supplementary Material: Expression and Biological Activity Analysis of Recombinant Fibronectin3 Protein in *Bacillus subtilis*

Chaozheng Lu, Guangxin Xu, Yin Tian, Zhiwei Yi * and Xixiang Tang *

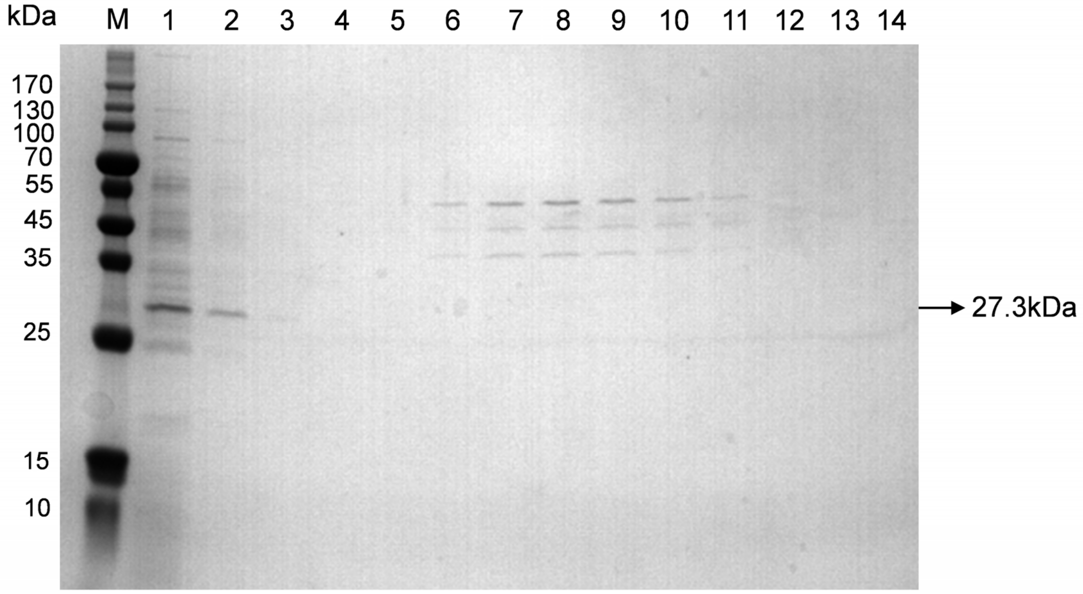

Figure S1. The fermentation supernatant underwent protein purification via nickel affinity chromatography. M: Protein marker; 1: Flow-through fraction; 2-5: The 1st-4th tubes of the eluent containing impurity proteins eluted with 3 mM imidazole in sequence; 6-14: The 1st-9th tubes of the eluent containing the target protein eluted with 50 mM imidazole in sequence.
